# Confining Fluorescent Probes in Nanochannels to Construct Reusable Nanosensors for Ion Current and Fluorescence Dual Gating

**DOI:** 10.3390/nano12091468

**Published:** 2022-04-26

**Authors:** Dan Zhang, Chunfei Wang, Changfeng Wu, Xuanjun Zhang

**Affiliations:** 1 MOE Frontiers Science Center for Precision Oncology, Faculty of Health Sciences, University of Macau, Macau 999078, China; yb97663@um.edu.mo (D.Z.); chunfeiwang@um.edu.mo (C.W.); 2 Department of Biomedical Engineering, Southern University of Science and Technology, Shenzhen 518055, China; wucf@sustech.edu.cn

**Keywords:** nanochannel sensor, dual gating, ion current, fluorescence, Hg(II), reusable

## Abstract

Here, we confined fluorescent probes to solid nanochannels to construct nanosensors, which not only significantly improved the reusability of the molecular probes, but also achieved ion current and fluorescence dual gating for more reliable detection. The combination of optical and electrical modalities can provide comprehensive spatiotemporal information that can be used to elucidate the sensing mechanism within the nanochannel. As a proof-of-concept experiment, fluorescein isothiocyanate (FITC)–hydrazine (N_2_H_4_) was selected to modify nanochannels for the effective detection of Hg^2+^. Based on spirolactam opening tactics, the system synergistically alters the surface charge and fluorescence intensity in response to Hg^2+^, establishing a dual open state of current and fluorescence. The newly prepared nanosensor exhibited a fast response (<1 min), high sensitivity, and selectivity towards Hg^2+^. Importantly, the nanodevice could be recovered by simple N_2_H_4_ treatment. Such sensing behavior could be used to implement optoelectronic dual-output XOR logical gates under the management of Hg^2+^ and N_2_H_4_. This strategy is anticipated to find broad applications in other nanochannel-based systems for various sensing applications used for monitoring of pollutants, food additives, and biomolecules.

## 1. Introduction

Inspired by biological protein channels, biomimetic solid-state nanochannels with ordered porous structures have received a large amount of research interest in the biochemical sensing field in recent decades [1,2,3,4,5,6]. Owing to their strong mechanical rigidity and flexible tunability in terms of geometry and surface modification [7,8,9,10,11,12,13], these nanochannels can be used as a promising skeleton to confine different types of probes, including small molecules and biomolecules (e.g., DNA, RNA, and proteins) [5,13,14,15]. Upon proper immobilization with the designed probes, targets can interact specifically with these functional molecules to change the nanochannels’ properties, such as their surface charge, wettability, and/or effective aperture [3,4,5,14,15,16,17]. Owing to the confining effect within nanochannels, these changes eventually trigger a highly sensitive response to transmembrane ion currents [18,19]. Additionally, the sensitive single electronic readout from picoampere to nanoampere is susceptible to nonspecific adsorption, obstruction of orifices, and/or different operation and equipment, increasing the likelihood of false-positive errors [20,21]. The lack of spatial visualization further blurs the actual response process inside the channel and erodes our ability to judge the corresponding reaction mechanism [22,23]. Monitoring fluorescence in nanochannels provides a direct and convenient way to assess spatial information. Although nanochannels can be lightened by attaching fluorescent dyes to nanochannel surfaces [24,25,26], this fluorophore is not involved in the sensing process, and does not reflect the reactivity of target analytes. Therefore, confining fluorescent probes into a nanochannel system can effectively fuse the complementary strengths of small-molecule fluorescent probes and nanochannels to achieve the current–fluorescence dual-signal monitoring of the analytes and improve the reusability of expensive probes. Additionally, benefiting from the unique confinement effect of the special microenvironment of nanochannels, the probe-functionalized sensor allows for the detection of lower concentrations and increases the detection sensitivity.

Mercury ions (Hg^2+^) are among the most toxic metal contaminants, and are widely distributed in solution matrices [27]. Mercury, in any form, can pose a serious and permanent threat to cell architecture function and human health by damaging the DNA, brain, kidneys, and digestive and central neurological systems—even in trace amounts [27,28,29]. The World Health Organization (WHO) and the US Environmental Protection Agency (EPA) have set the maximum allowable limits of Hg^2+^ in drinking water to 30 and 10 nM, respectively [30,31]. To date, abundant mercury-indicating techniques in water samples have been reported, typically including liquid molecule fluorescence sensors and solid-state nanochannel platforms. Among them, the fluorescence probe for Hg^2+^ based on a fluorescein derivative promises fruitful applications due to its fast response time and visual monitoring. Its chemistry has also been well documented [32]. When spirolactam formation (cyclization) between fluorescein and N_2_H_4_ occurs, the emission intensity of fluorescein hydrazide is very low. After binding to Hg^2+^ ions, Hg^2+^ can induce the spiro ring to open and form fluorescein, resulting in a dramatic increase in the green fluorescence. However, the liquid phase fluorescent assay has limited ability for cyclic detection due to their easy photobleaching, difficulty to separate, and poor recoverability [33,34]. In addition, all reported nanochannel sensors towards Hg^2+^ are based on the specific interaction between Hg^2+^ and thymine (T)-rich DNA probes to form stable T–Hg^2+^–T complexes [25,26,35,36]. This conformational shift triggers an effective pore size change to control the transmembrane current. However, the formation of a double-stranded rigid structure requires a reaction time of several hours. Its reaction mechanism in the channel also lacks an intuitive verification method, such as fluorescence signals [24,36,37]. Accordingly, it is imperative to exploit fast, reusable, and reliable sensors for the analysis of Hg^2+^ in aqueous samples. The transfer of effective spiro-ring switch events into solid nanochannels may easily enable the reuse of probes and dual-mode monitoring of current and fluorescence.

**Figure 1:**
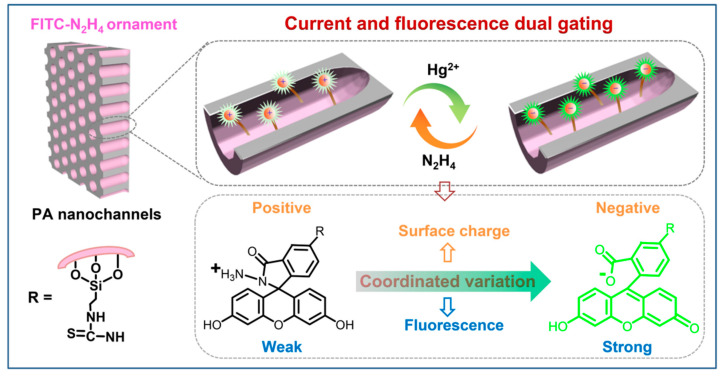
Schematic of the reusable Hg^2+^-responsive nanochannel sensor for current and fluorescence dual gating. The detector is based on FITC–N_2_H_4_-modified alumina nanochannels. The FITC–N_2_H_4_ probe is reversible upon alternative treatment with Hg^2+^ and N_2_H_4_.

Herein, as a prototype example, we confined spiro fluorescent probes in solid nanochannels to construct a reusable Hg^2+^ nanosensor with dual gating of ionic current and fluorescence (Figure 1). After achieving stepwise attachment of fluorescein isothiocyanate (FITC) and hydrazine (N_2_H_4_) to the channels, the FITC–N_2_H_4_ probe in the closed spirolactam form showed a positive charge and weak fluorescence. At this point, the nanochannels presented a dual-closed gating status of current and fluorescence. Upon detection of Hg^2+^, the probe would isomerize to a ring-opening form, leading to a negatively charged state and fluorescence enhancement at the same time. Therefore, the original optoelectronic dual-closed gating was converted into dual-open gating. Importantly, the probe can be readily recycled to the ring-closed form by reacting with N_2_H_4_. This feature endows the nanochannel sensor with reversible switching via the alternative stimulation of Hg^2+^ and N_2_H_4_. It is therefore used to support the development of current and fluorescence dual-output XOR logical gates. Our nanochannel platform allows for mutual verification of the detection results obtained from the dual electro-optical modes, effectively improving the accuracy and reliability of the detection.

## 2. Materials and Methods

### 2.1. Materials

Perchloric acid (HClO_4_), phosphoric acid, chromium trioxide, and potassium chloride were purchased from Beijing Chemical Works (Beijing, China). Oxalic acid (H_2_C_2_O_4_) was purchased from Aladdin (Shanghai Aladdin Biochemical Technology Co., Ltd, Shanghai, China). Fluorescein, hydrazine hydrate (N_2_H_4_·H_2_O), hydrogen peroxide (H_2_O_2_), potassium hydroxide (KOH), hydrochloric acid (HCl), mercury(II) nitrate dihydrate (Hg(NO_3_)_2_·2H_2_O), and other metal salts were purchased from Sigma-Aldrich (Sigma-Aldrich, Inc., St. Louis, MO, USA). (3-Aminopropyl) triethoxysilane (APTES) and fluorescein isothiocyanate (FITC) were purchased from Dieckmann (Dieckmann Technology Development Co., Ltd., Shenzhen, China). Silver wire, platinum wire, stainless steel sheets, and aluminum foil (99.999% pure) were purchased from Trillion Metals Co., Ltd (Beijing, China). All chemicals were supplied by commercial distributors and used without further purification.

### 2.2. Characterization

The surface morphologies of the nanotube-shaped alumina nanochannels at the base and tip sides, as well as their cross-sections, were characterized using an FEI Quanta FEG 250 environmental scanning electron microscope (SEM). Chemical analysis of the composition and elements of Al_2_O_3_ nanochannels upon the modification of each step was conducted using an ESCALAB 250Xi XPS (Thermo Fisher Scientific, Waltham, MA, USA). Water contact angles (CAs) were measured using an OCA40 contact angle system (DataPhysics, Filderstadt, Germany). Fluorescence spectroscopy and confocal images were performed using a HORIBA FluoroLog-3 Modular Spectrofluorometer (Horiba Instruments, Acal BFi, Eindhoven, The Netherlands) and a Carl Zeiss LSM710 confocal microscope (Carl Zeiss Pty Ltd., Oberkochen, Germany), respectively. The excitation wavelength was set to 488 nm.

After the attachment of FITC on the nanochannel surface, fluorescence spectroscopy (XPS) analysis revealed a new S2p peak at 165 ev, which was due to the sulfur element of FITC. The decrease in the CA value indicated a more hydrophilic surface. The ionic current in the *I*—*V* analysis increased in the reverse direction, indicating a negative surface charge generated by the FITC. In fluorescence spectroscopy, the distinct peak at 518 nm is the characteristic emission peak of FITC. In laser confocal imaging, green fluorescence was observed in the cross-section of the nanochannel, directly indicating that the FITC had successfully attached to the interior of the nanochannel. However, after being treated with hydrazine, the content of nitrogen elements increased, which was attributed to the cyclization reaction of FITC with hydrazine. The increase in CA indicated a decrease in surface hydrophilicity. In the *I–V* analysis, the ionic current decreased and the direction reversed again, indicating the positive surface charge from N_2_H_4_. In fluorescence spectroscopy, the characteristic fluorescence peak from FITC at 518 nm was significantly decreased. In laser confocal imaging, the original green fluorescence was not observed. These results suggested that the further conjugation of N_2_H_4_ with FITC in the nanochannels triggered spirolactam formation. In a word, based on XPS, CA, *I–V* curves, fluorescence spectroscopy, and confocal images, we can clearly demonstrate the successful modification of FITC and N_2_H_4_ on the surface of the nanochannel.

### 2.3. Preparation of the Nanotube-Shaped Alumina Nanochannels

The nanotube-shaped alumina nanochannels were prepared via two-step anodization combined with etching of the aluminum substrate and the barrier layer [38,39]. Firstly, Al foil (99.999% pure) was cleaned with acetone, ethanol, and Milli-Q water (18.2 MΩ) successively. Then, the resulting Al foil was used as the anode to conduct electropolishing in a mixed solution of HClO_4_ and ethanol (1:4 in volume ratio) at 5 °C under a voltage of 17 V for 7 min to achieve a mirror-finished surface, while a graphite plate was used as the cathode. Subsequently, the first anodization was carried out in a 0.3 M oxalic acid solution at 5 °C for 2 h. However, the obtained disordered porous oxide layer needed to be peeled off the Al substrate in a mixing solution containing 6 wt% phosphoric acid and 3.5 wt% chromic acid at 90 °C for 2 h. Then, the residual Al substrate with hemispherical concaves was re-anodized for 6 h under the same conditions as the first anodization to grow highly ordered Al_2_O_3_ porous arrays. To expose the barrier oxide layer against the porous Al_2_O_3_ arrays, sodium hydroxide and saturated copper dichloride solution were continuously used to etch away the bottom of the Al substrate. Finally, the accurate pore-opening procedure of the barrier layer in 6 wt% H_3_PO_4_ was the key to regulating the transmembrane ion transport properties. The adjustment for pore size was closely dependent on the reaming time. Morphologies at the barrier layer side after the etching were observed via SEM.

### 2.4. Sequential Immobilization of APTES, FITC, and N_2_H_4_ on the Nanochannels

The as-prepared nanotube-shaped Al_2_O_3_ nanochannels were firstly boiled in hydrogen peroxide for 1 h to introduce more hydroxylate groups onto the inner surface of the nanochannels. The resulting nanochannels were immersed in an ethanol solution of APTES (16 vt%) for 1 h to enrich the pore surface with amino groups. Such free amino groups were used for covalently immobilizing FITC through the formation of thiourea bonds overnight. With that, the FITC-functionalized nanochannels carrying carboxylic groups further reacted with N_2_H_4_ (25 mM) overnight in ethanol solution. All of the membranes were washed with ethanol solution and deoxygenated Milli-Q water (18.2 MΩ) before *I–V* measurement.

### 2.5. Ionic Current Measurements

The ionic current and rectification characteristics of nanochannels under various states of modification were characterized by investigating transmembrane *I–V* curves at varied scanning voltages from −2 to +2 V, which were provided by a Keithley 6487 picoammeter (Keithley Instruments, Cleveland, OH, USA). Aqueous potassium chloride solution at 1 mM was chosen as the electrolyte. The nanochannel membrane was mounted between two chambers of the electrochemical cell, in each of which one Ag/AgCl electrode was vertically installed to produce the transmembrane potential driving electrolytes across the nanochannels. The base side of the alumina nanochannels was determined to provide the positive potential, while the tip side was found to provide the negative potential. All of the tests were conducted at room temperature. The response measurement was conducted on the PA channel after being treated with Hg^2+^ for 15 min, unless otherwise specified.

## 3. Results and Discussion

### 3.1. Characterization of the Nanochannels

**Figure 2:**
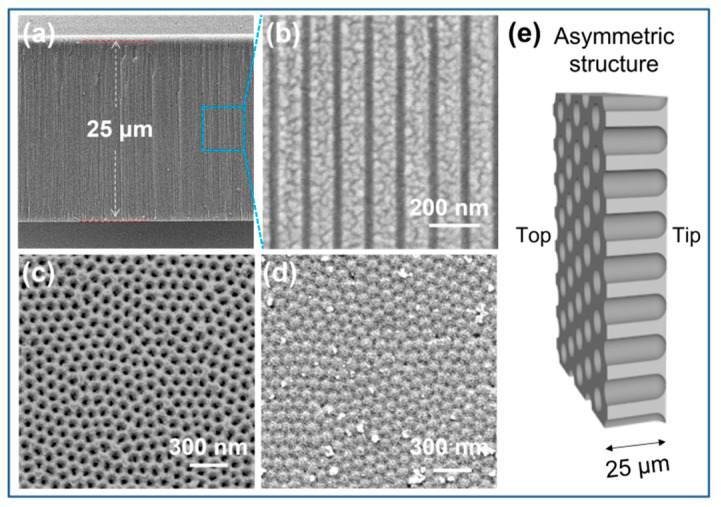
SEM images from (**a**,**b**) the cross-sectional view of the alumina nanochannel at low and high magnifications, respectively, and from (**c**,**d**) the opening base and tip sides of the Al_2_O_3_ barrier layer, respectively. (**e**) As a whole, the nanochannels present an asymmetric structure.

The porous alumina nanochannels were fabricated via a two-step anodization method combined with an etching procedure of the aluminum substrate and the barrier layer, as reported in [38,39]. The entire nanochannel exhibited an asymmetric geometric skeleton similar to that of a nanotube. As investigated by scanning electron microscopy (SEM), the thickness of the resultant alumina nanochannels was estimated at 25 µm (Figure 2a). The diameter of the intermediate parallel channels was approximately 35 nm (Figure 2b), which was equivalent to the aperture at the opening base side (Figure 2c). Conversely, at the tip side, a close-packed semispherical hexagon on the barrier layer could be observed (Figure S1a). This impeded the smooth transport of ions across the membrane. Therefore, to increase the membrane permeability while maintaining asymmetric nanotube traits, the pore-opening procedure of the barrier layer was precisely controlled by adjusting the chemical etching time (Figure 2d and Figure S1b,c). The results show that the diameter of the tip side increased appreciably with increasing etching time. As a result, the structure of the nanochannel membrane was transformed from asymmetric to symmetric. A typical SEM observation (Figure 2d) of the bottom surface showed the appearance of tiny, subnanometer-wide cracks after 10 min of etching, which could provide a confined transmission path for ions. In this case, maximizing the asymmetric morphology (Figure 2e) would lead to optimal ion transport behavior, including ionic conduction and ionic rectification.

### 3.2. Construction of the Functionalized Nanochannel Sensor

**Figure 3:**
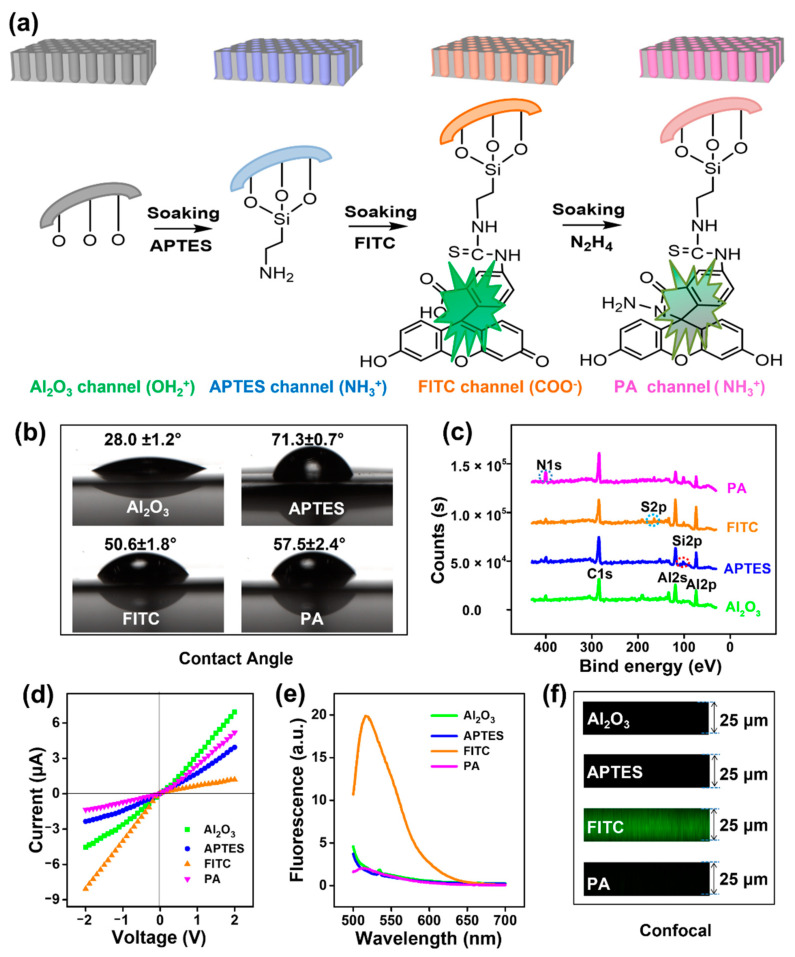
Characterization of the nanochannel modification process: (**a**) The construction evolution for Hg^2+^-gated nanochannels from Al_2_O_3_ to APTES, FITC, and probe-modified alumina (PA) nanochannels by APTES, FITC, and N_2_H_4_ soaking treatment, in sequence. In this process, the surface charge and fluorescence characteristics of the nanochannels were changed; the characterizations for these channels via (**b**) wettability, (**c**) elemental composition studied by XPS, (**d**) *I–V* curves, (**e**) fluorescence spectra, and (**f**) confocal imaging.

Given the ring-opening recognition behavior of spirolactam towards Hg^2+^, the FITC–N_2_H_4_ probes were selected to be covalently grafted onto the inner surface of the alumina nanochannels by stepwise modifications (Figure 3a). (3-Aminopropyl) triethoxysilane (APTES), as a bifunctional molecular glue, can effectively conjugate the FITC to the Al_2_O_3_ channels. The triethoxysilane group can be bonded to the Al_2_O_3_ surface via the Al–O–Si bond, while the amino group can conjugate with fluorescein isothiocyanate via thiourea [40]. In the next step, the Hg^2+^-response moiety, -CONNH_2_, was formed in situ by simple N_2_H_4_ treatment. The final probe-functionalized alumina (PA) nanochannels were thoroughly washed with ethanol to remove any unconjugated free molecules. Throughout the process, from the Al_2_O_3_ channel to the APTES channel, as well as the FITC channel and the PA channel, the surface charge was transformed by positively charged hydroxyl groups (-OH_2_^+^), positively charged amino groups (-NH_3_^+^), negatively charged carboxyl groups (-COO^−^), and positively charged -NH_3_^+^. Meanwhile, the probes grafted to the channels showed fluorescence quenching due to the spirolactam closing.

Successful stepwise modifications were confirmed by contact angle (CA) measurement and X-ray photoelectron spectroscopy (XPS) (Figure 3b,c). As expected, the attachment of APTES and FITC on the nanochannel surface resulted in a new Si2p peak at 103 eV and an S2p peak at 165 eV [41,42]. After being treated with hydrazine, the content of nitrogen elements in the FITC-modified nanochannels increased. This was due to the cyclization reaction of FITC with hydrazine. The modification was further characterized by typical transmembrane current–voltage *(I–V)* responses, which were recorded in a two-compartment electrochemical reservoir filled with 1 mM electrolytic KCl solutions (Figure S2). The change in wettability had an effect on ion conduction. The change in the polarity of the surface charge resulted in a reversal of the direction of current rectification. As shown in Figure 3d, before modification, the channel surface was positively charged (-OH_2_^+^) in neutral electrolytes because the isoelectric point (IEP) of the terminal hydroxyl groups on alumina was approximately 8.7 [43]. In this case, more chloride would be attracted into asymmetric nanoconfined Al_2_O_3_ spaces. These chloride anions preferred to flow from the tip to the base side at a positive voltage, thus bringing about an ionic rectification behavior—higher currents at positive voltages than at negative voltages. Compared with other channels, the highest current conduction at +2 V for Al_2_O_3_ should be attributed to its superhydrophilic nature, with CAs of approximately 28°. After modification by APTES, the current rectification direction of the alumina nanochannels did not change, as the inner surface rich in amine groups was shown to be positively charged. However, ion conduction of the APTES channel was significantly reduced due to its enhanced hydrophobicity, which hindered ion transport across the membrane to some extent [40]. The introduction of FITC chromophores on the channel triggered an immediate reversal of the rectification characteristics and augmented the current conduction at −2 V. This was attributed to the deprotonation of -COOH groups from FITC, which formed a negatively charged hydrophilic surface. Finally, the electronegative carboxyl groups were replaced by amino groups through subsequent N_2_H_4_ treatment, and the current rectification direction was reversed again. Accordingly, the rectification properties provided more intuitive evidence for the successful modification and the transition in surface charge (Figure S3).

In addition, fluorescence spectroscopy (Figure 3e) and laser confocal imaging (Figure 3f) in view of spatial information were employed to track the modification progress of probe molecules. FITC, as a fluorescein derivative, has strong green fluorescence. The excitation wavelength was 488 nm. As a result, the green fluorescence peak at 518 nm appeared after FITC was attached to the surface of the nanochannel, while the original AAO and APTES channels had no fluorescence peaks (Figure 3e). By cross-sectional confocal imaging, obvious green fluorescence could be observed from the FITC nanochannels (Figure 3f). These intuitive fluorescence signals clearly indicated that FITC was successfully attached to the inner surface of the nanochannel, whereas the further conjugation of N_2_H_4_ with FITC triggered spirolactam formation, resulting in significant declines in the fluorescence intensity [44]. As shown in Figure 3e,f, the emission peak and green fluorescence intensity were dramatically reduced after N_2_H_4_ treatment. The changes in spatial fluorescence indicate that cyclization of FITC in the nanochannels occurs readily with N_2_H_4_.

### 3.3. Current and Fluorescence Dual Gating and Renewability of the Nanosensor

**Figure 4:**
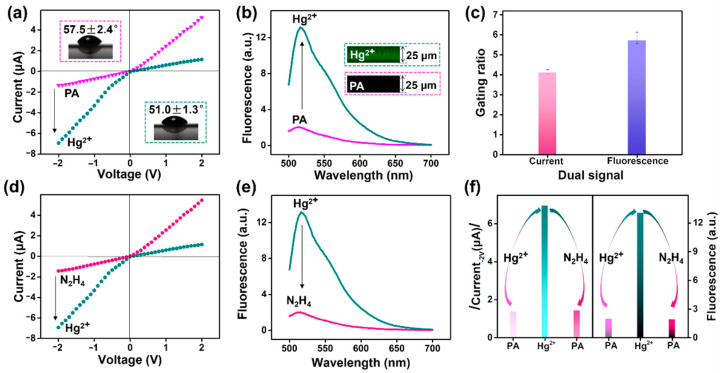
Current and fluorescence response of PA nanochannels to Hg^2+^ and N_2_H_4_: (**a**) *I–V* curves of PA nanochannels upon the treatment with 10 μM Hg^2+^; the insets represent corresponding wettability change by contact angles (CAs). (**b**) Fluorescence response of PA nanochannels upon the treatment with 10 μM Hg^2+^; the insets represent corresponding fluorescence images by laser scanning confocal microscopy (LSCM). (**c**) Hg^2+^-activated gating ratio of the current signal and fluorescence signal of the PA nanochannels. (**d**) *I–V* curves of Hg^2+^ channels upon the N_2_H_4_ treatment. (**e**) Fluorescence response of Hg^2+^ channels upon the N_2_H_4_ treatment. (**f**) Corresponding change contrast of current (absolute value at −1 V) and fluorescence upon continuous stimulation of Hg^2+^ and N_2_H_4_.

We investigated whether the probe molecules confined in nanochannels could trigger fluorescence and current dual gating. As shown in Figure 4a, a rectification behavior was observed in asymmetric PA nanochannels, with a lower conductance at −2 V. This is because in the confined space of protonated NH_3_^+^, Cl^−^ was selectively transported through the channel from its tip side. However, under stimulation with Hg^2+^ (10 μM), the ionic conductance at −2 V immediately increased to −6.94 μA from −1.36 μA, and a reversal of the rectification characteristics occurred. The wettability of the channel membrane also increased with the ionic conductance (the CA decreased from 57.5 ± 2.4° to 51.0 ± 1.3°). This might have been due to the specific catalytic reaction between the Hg^2+^ and the CONNH_2_ moiety of the fluorescent probe, which can trigger the ring opening of spirolactam and the charge transformation from positive (NH_3_^+^) to negative (COO^−^). In this case, more K^+^ cations could be attracted into the channels, resulting in an inversion of current rectification and enhancement in current conduction at −2 V. Meanwhile, the recovery of the FITC component on the channel surface also intrinsically turned on fluorescence, as revealed by both fluorescence spectra and fluorescence confocal imaging (Figure 4b). The fluorescence change trend in response to Hg^2+^ in the channel system was consistent with that of the free fluorescein–N_2_H_4_ molecule (Figure S4) in the solution system (Figure S5). The detailed response mechanism is shown in Figure S6. As reported, the Hg^2+^ can first chelate N and O on the hydrazone group of the probe to form a fluorescein–N_2_H_4_–Hg^2+^ complex. Then, the metal–ligand complex undergoes hydrolysis of the amide bond to assist the ring opening, resulting in the formation of fluorescein [42,45,46]. The entire process triggers a conformational transformation from fluorescein spirolactam to ring-opening fluorescein. This ring-opening phenomenon was also supported by corresponding hydrogen nuclear magnetic resonance spectrum (^1^H NMR) analysis (Figure S7). In short, after Hg^2+^ treatment, the closed-ring FITC–N_2_H_4_ probe in the liquid system was converted into an open-ring FITC component with enhanced fluorescence. When the probes were introduced into the solid nanochannels, the Hg^2+^-induced ring-opening behavior not only resulted in the enhancement of the fluorescence signal, but also caused an increase in ionic current due to the reversal of the surface charge from NH_3_^+^ to COO^−^. That is, the nanochannel provides an ideal vehicle for this fluorescent probe, enabling simultaneous conversion of current and fluorescence signals. The photoelectric double-check strongly manifested the ring-opening mechanism rather than the nonspecific adsorption of targets. Importantly, the nanosensor device had excellent stability. As shown in Figure S8a,b, the responsiveness of the nanosensor to Hg^2+^ (both current and fluorescence) did not deteriorate even after a year of placement.

Gating ratios as an expression of current and fluorescence responses to Hg^2+^ were presented to discuss the performance of the nanosensor. Here, the ionic current gating ratio is defined as ((I − I_0_)/I_0_, −2 V), where I and I_0_ are ionic currents at −2 V in the presence and absence of Hg^2+^, respectively. The fluorescence gating ratio is defined as ((F − F_0_)/F_0_, 518 nm), where F_0_ and F are the fluorescence intensity at 518 nm before and after Hg^2+^ stimulation, respectively. Notably, the current and fluorescence gating ratios shown here were 4.1 and 5.7, respectively (Figure 4c). Accordingly, synergistic current and fluorescence gating devices were successfully built. Importantly, the ion current (Figure 4d) and fluorescence signal (Figure 4e) reversibly returned to a lower value after the reaction with N_2_H_4_. The combined results demonstrated that both current and fluorescence first increased after Hg^2+^ detection, and then decreased to their original values after N_2_H_4_ treatment (Figure 4f). In other words, the PA nanochannel sensor could be restored to its initial state under the alternating treatment with Hg^2+^ and N_2_H_4_.

**Figure 5:**
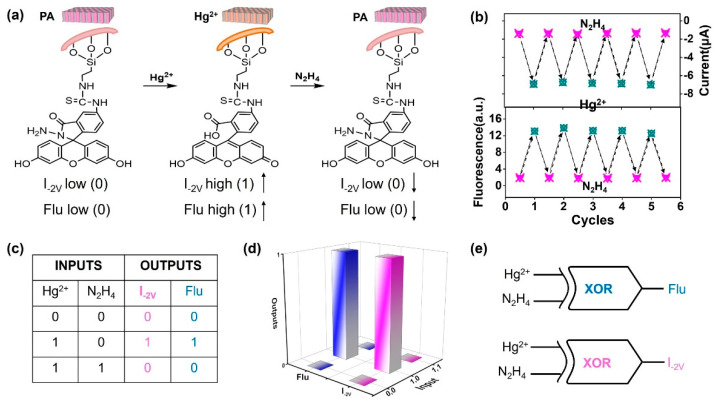
Recyclability of PA nanochannels and dual-output XOR gates: (**a**) Schematic diagram of PA nanochannels for Hg^2+^ sensing and the subsequent reversible recovery by in situ N_2_H_4_ treatment. (**b**) Current at −2 V (upper) and fluorescence (lower) cycles upon alternative stimulation of Hg^2+^ and N_2_H_4_, indicating Hg^2+^-response stability and recyclability of the PA nanochannel system. (**c**) Truth tables. (**d**) Corresponding logic functions scheme. (**e**) Dual-output XOR gates of the PA nanochannel system, obtained by continuously adding the Hg^2+^ and N_2_H_4_ as dual inputs.

Based on the recovery scenarios (Figure 5a), the reusability and stability of the nanosensor were investigated by monitoring both current traces at −2 V and fluorescence intensity at 518 nm upon multiple alternating treatments with Hg^2+^ and N_2_H_4_ (Figure 5b) [44]. Even after five cycles, neither the current nor the fluorescence showed signs of faltering. Such robustness greatly improved the practicability of the nanosensor. Using the principle of the PA nanochannels (Figure 5a), the system was further exploited to realize two XOR logical gates by successively bringing in Hg^2+^ and N_2_H_4_. This demonstrates the operational availability of the nanochannel system for current and fluorescence dual gating with reliable sensing. We considered the additions of Hg^2+^ and N_2_H_4_ as two input signals, and their presence and absence as “1” and “0”, respectively. For the current output, we assigned “low conduction at −2 V” as “0” and “high conductance at −2 V” as “1”. Similarly, for the fluorescence output at 518 nm, we assigned “weak fluorescence” as “0” and “strong fluorescence” as “1”. The truth table and corresponding logic scheme (Figure 5c,d) revealed that these logic operations were in accordance with a two-input and two-output excluded OR (XOR) gate (Figure 5e) [47,48,49]. Without (0,0) or with (1,1), and the simultaneous addition of Hg^2+^ and N_2_H_4_, the surface composition of the PA channel remained as the hydrazine component. Thus, both the current and the fluorescence stayed at their low levels, producing the “0,0” dual-output signal. This suggests that the PA system is reversible for Hg^2+^ sensing. In contrast, the introduction of Hg^2+^ (1,0) alone led to the ring opening and exposure of the FITC component. In this case, ionic current at −2 V and fluorescence were significantly enhanced, featuring the “1,1” photoelectric dual output. The dual XOR logic gates in the Hg^2+^ sensor allow for higher reliability than single logic does. In a word, solid-phase attachment of fluorescent probes to the nanochannels not only offers the advantage of optical-electric bivariate detection, but also makes the sensor readily reusable. Compared to previous liquid-phase fluorescent probes or DNA-based nanochannel sensors, our nanochannel platform allows for reuse of the fluorescent probe, and also provides dual-output gates for current and fluorescence [25,32]. These features endow the monitoring system with practicality and reliability.

### 3.4. Evaluation of Response Performance for Hg^2+^

**Figure 6:**
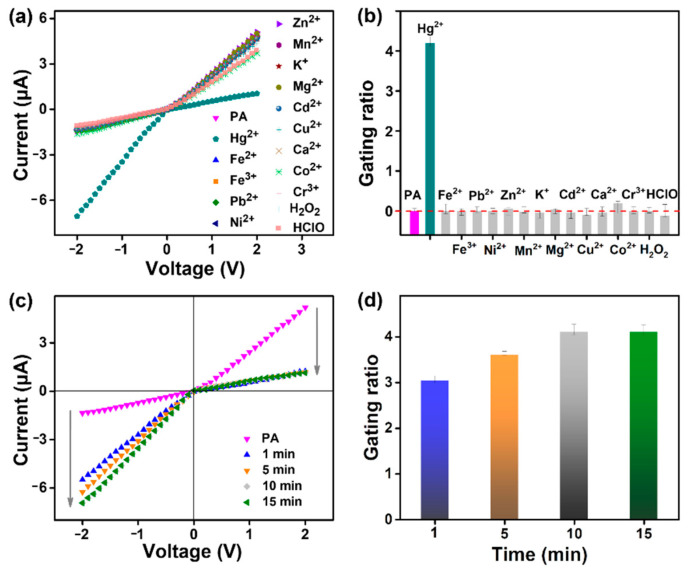
Selectivity and response time of the PA nanochannels to Hg^2^^+^ detection: (**a**) *I–V* curve performance in different analytes, and (**b**) the corresponding gating ratios for different analytes. (**c**) *I–V* curve performance with response time, and (**d**) the corresponding gating ratio.

The high selectivity, sensitivity, and fast response of the nanochannel platform are desirable in effective analysis. *I–V* features were adopted to assess these important performance factors. To investigate the selectivity to Hg^2+^, the nanosensor was treated with miscellaneous kinds of components (10 μM), including metal ions (Hg^2+^, Fe^2+^, Fe^3+^, Pb^2+^, Ni^2+^, Zn^2+^, Mn^2+^, K^+^, Mg^2+^, Cd^2+^, Cu^2+^, Ca^2+^, Co^2+^, and Cr^3+^) and H_2_O_2_ as well as HClO. As observed in Figure 6a, the ion current at −2 V increased from −1.36 μA to −7.06 μA only after activation by Hg^2+^. After treatment with other metal ions and reactive oxygen species, the ionic current remained unchanged. Thus, the PA system showed a higher current-gating ratio (4.2) under Hg^2+^ stimulation than the other analytes (Figure 6b). In contrast, there were no significant current and fluorescence changes in the Al_2_O_3_, APTES, and FITC channels after 10 μM Hg^2+^ treatment (Figure S9a–c). It is exciting that the PA nanosensors still exhibited a high current response to Hg^2+^ in the dye-doped matrix, such as solution samples containing diversified dyes (Figure S10a) or fluorescent trackers for cell studies (Figure S10b). As long as there was no Hg^2+^ stimulation, fluorescent dyes—either alone or in combination—did not cause a change in the PA channel current. Instead, once 10 μM Hg^2+^ was introduced into this system doped with dyes, current variation in the magnitude and direction of the PA channels could be observed immediately. These merits are expected to enable this nanosystem to monitor Hg^2+^ in complicated environments, such as the printing and dyeing industries.

In addition, the scanning *I*–*V* curves were investigated based on incremental response time. As shown in Figure 6c, the PA nanochannels’ ionic current at −2 V was ≈ −1.36 μA. When 10 μM Hg^2+^ was applied, the current significantly increased to −5.49 μA within 1 min, and leveled off at −6.93 μA over 10 min. Even when the response was extended to 15 min, the ionic current did not increase further. Reversal of the current direction was attributed to a rapid mutation in surface charge from positive to negative. After the addition of Hg^2+^ for 1 min, the gating ratio immediately changed to 3.0, and the gate switched from the OFF to the ON state. Then, the ratio slightly increased to 3.6 at 5 min, and eventually stabilized at 4.1 over 10–15 min to reach the complete ON state (Figure 6d). Clearly, the probe-functionalized nanosystem was endowed with a fast response to Hg^2+^. Such molecular ring opening is faster than the formation of T–Hg^2+^–T in the DNA-based nanochannels [36]. 

**Figure 7:**
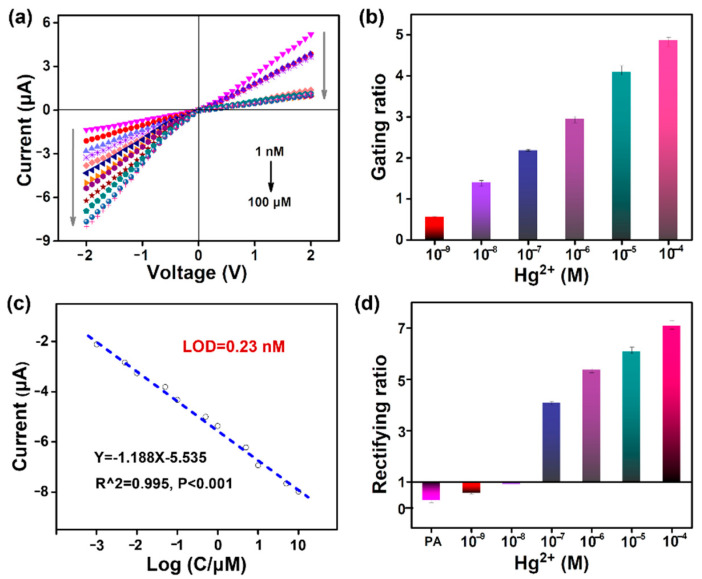
Sensitivity of the PA nanochannels to Hg^2^^+^ detection: (**a**) *I–V* curve response performance of the PA nanochannels with Hg^2+^ concentration; (**b**) the corresponding gating ratio, (**c**) detection sensitivity, and (**d**) rectifying ratio.

Finally, the *I–V* curve response was investigated under a wide range of Hg^2+^ concentrations (Figure 7a). As the Hg^2+^ concentration increased from 1 nM to 100 μM, the ionic current at −2 V gradually rose. This suggests that the current gate switched from the OFF state to the ON state (Figure 7b). Meanwhile, the fluorescence versus Hg^2+^ concentration could also be monitored via fluorescence spectroscopy (Figure S11a) and confocal imaging (Figure S11b). As the Hg^2+^ concentrations increased, the fluorescence gating switched from the OFF state to the ON state (Figure S11c). Importantly, the plot of current versus the logarithm of the Hg^2+^ concentration (Log C, C = 1 nM–10 mM) showed a good linear correlation. As shown in Figure 7c, the typical calibration curve was Y = −1.188 X − 5.535 (R^2^ = 0.995; Y and X represent the ionic current and Log C, respectively). On the grounds of a classical 3 SD/L method, where SD is the standard deviation of the blank and L is the slope of the calibration curve [5], the detection limit of this nanochannel system was estimated to be 0.23 nM. The corresponding rectifying ratio calculated in Figure 7d describes the response characteristics during Hg^2+^ recognition more intuitively. The rectifying ratio showed less variation, in the range of 10^−9^–10^−8^ M Hg^2+^. However, when the concentration increased to 10^−7^ M, the rectifier suddenly increased to greater than 1, implying an inversion of the surface charge polarity. As the concentration of Hg^2+^ rose from 10^−7^ to 10^−4^ M, the rectifying ratio continued to increase due to the efficient ring-opening reaction producing increasing amounts of -COOH. On the whole, the newly developed nanochannel sensor had good performance in the quantitative analysis of Hg^2+^. Additionally, the designed sensing platform was successfully used for the determination of Hg^2+^ in lab wastewater (Figure S12), demonstrating its applicability in the analysis of real water samples.

## 4. Conclusions

To conclude, we developed a general strategy for confining fluorescent probes in nanochannels to construct a reusable nanosensor with the dual gating of fluorescence and current. This union of temporal and spatial management allows for the mutual validation of specific recognition processes, thereby enhancing the detection reliability. In this fabrication, the FITC–N_2_H_4_ fluorescent probes were immobilized in the inner surface of the channel. Upon Hg^2+^ activation, current and fluorescence synchronously switched from the OFF state to the ON state as the result of the smooth cooperative effort of surface charge reversal and fluorescence intensity enhancement by Hg^2+^-catalyzed spiro-ring opening. In turn, the nanodevice could be restored in situ to the closed state by simple N_2_H_4_ treatment. Under alternating stimulation with Hg^2+^ and N_2_H_4_, the nanosensor was found to exhibit excellent reversibility and stability, which can be exploited to support current and fluorescence dual-output XOR logical gates. Moreover, this Hg^2+^-gating system exhibited a fast response within 1 min, a good sensitivity with a detection limit of 0.23 nM, and a high selectivity even in a dye-doped matrix. Overall, this strategy for confining fluorescent probes to nanochannels is versatile, and paves the way for the creation of a reusable and reliable photoelectric sensor.

## Data Availability

The data presented in this study are available upon request from the corresponding author.

## Supplementary Materials

The following supporting information can be downloaded at: https://www.mdpi.com/article/10.3390/nano12091468/s1, Figure S1: SEM images of the tip side from an alumina barrier layer at different chemical etching times; Figure S2: Schematic representation of the experimental setup for the *I*-*V* measurements; Figure S3: Ionic-current-rectifying ratio characteristics of the bare Al_2_O_3_, APTES, FITC, and PA nanochannels; Figure S4: The synthesis of spiro-form fluorescein hydrazide by the reaction of fluorescein with hydrazine hydrate; Figure S5: Liquid-phase fluorescence detection; Figure S6: Schematic representation of a possible mechanistic pathway of the catalytic reaction of Hg^2+^ on the non-fluorescent fluorescein–N_2_H_4_ probe to form highly fluorescent fluorescein; Figure S7: The ^1^HNMR spectra of the fluorescein–N_2_H_4_ probe in the absence and presence of Hg^2^+; Figure S8: Current and fluorescence response to Hg^2+^ of PA nanochannels after being kept out of the light for months; Figure S9: I–V properties of the Al_2_O_3_, APTES, and FITC nanochannels before and after Hg^2+^ stimulation; Figure S10: I–V curve response performance of PA nanochannels for indicating Hg^2+^ in a complex matrix containing diversified chromophore dyes or cell-tracking dyes; Figure S11: Fluorescence spectra, confocal imaging, and corresponding fluorescence gating ratios of the PA nanochannel system obtained in the presence of different concentrations of Hg^2^+; Figure S12: The applicability of the PA nanochannel system for the determination of Hg^2+^ in real-life samples.
