# Supplementary material for: Confining Fluorescent Probes in Nanochannels to Construct Reusable Nanosensors for Ion Current and Fluorescence Dual Gating
Source: Nanomaterials (Basel). 2022 Apr 26;12(9):1468. doi: 10.3390/nano12091468 (PMC9101493; doi:10.3390/nano12091468)
Supplement: Supplementary file 1 [file nanomaterials-12-01468-s001.zip › nanomaterials-1642427-Supplementary.pdf]

Supplementary Information

Confining Fluorescent Probes in Nanochannels to Construct Reusable Nanosensors for Ion current and Fluorescence Dual Gating

Dan Zhang [1], Chunfei Wang [1], Changfeng Wu [2] and Xuanjun Zhang [1,*]

[1] MOE Frontiers Science Center for Precision Oncology, Faculty of Health Sciences, University of Macau, Macau 999078, China; yb97663@um.edu.mo (D.Z.); chunfeiwang@um.edu.mo (C.W.)
[2] Department of Biomedical Engineering, Southern University of Science and Technology, Shenzhen 518055, China; wucf@sustech.edu.cn
* Correspondence: xuanjunzhang@um.edu.mo

Supplementary Figures

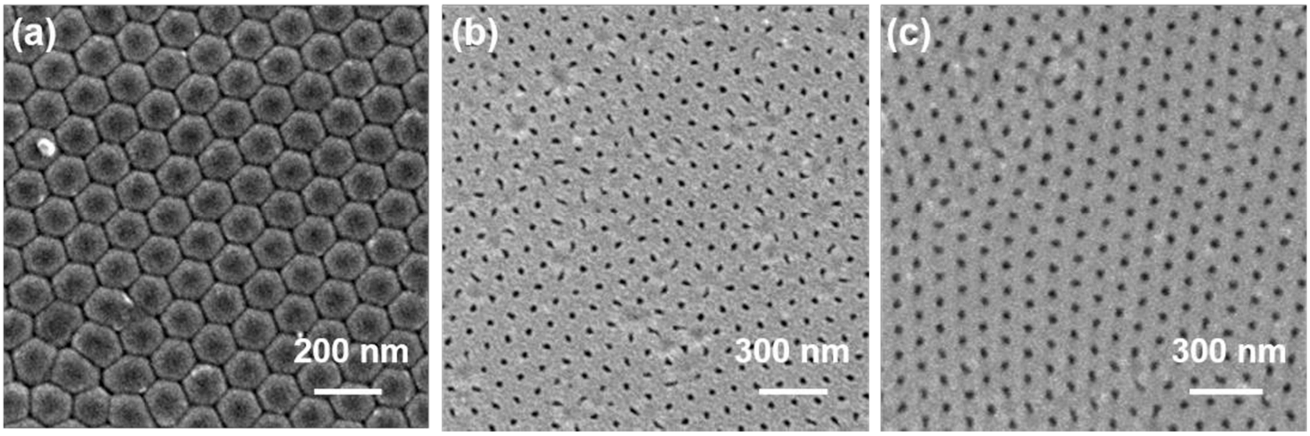

Figure S1. SEM images of the tip side from an alumina barrier layer when different chemical etching times of (a) 0, (b) 20, and (c) 30 min. The pore geometry evolution of the tip view over time can be observed from the sealed hemispherical dome state to opened porous state, resulting in the entire alumina nanochannels exhibiting the microstructural transformation from asymmetry to symmetry. In this case, the ionic transmembrane transportation behavior including rectification and selectivity characteristics will deteriorate accordingly.

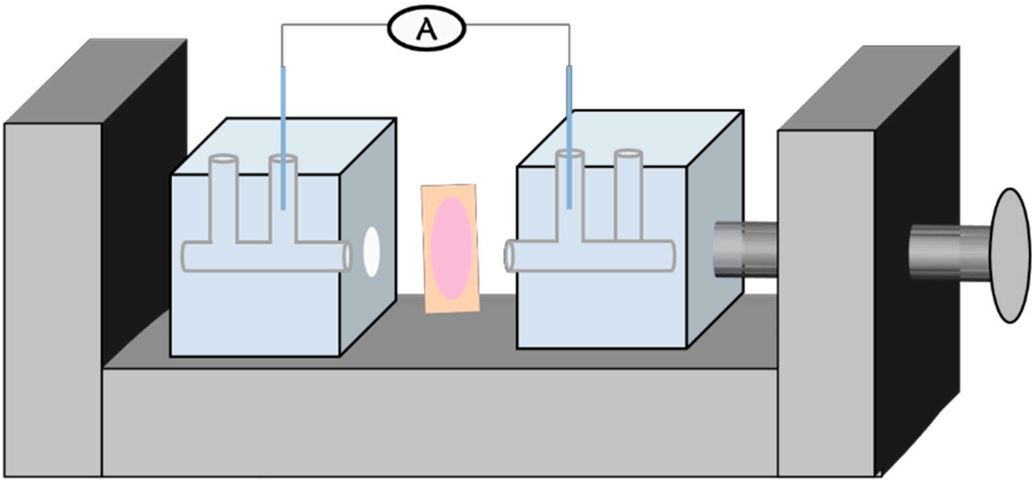

Figure S2. Schematic representation of the experimental setup for the *I-V* measurements. The nanochannel membrane was sandwiched between the two halves of the electro- chemical reservoir, which was injected with KCl electrolyte solution at a concentration of 1 mM. Place one Ag/AgCl electrode connected with a Keithley 6487 picoammeter on each side of the nanochannels' membrane to apply the transmembrane potential.

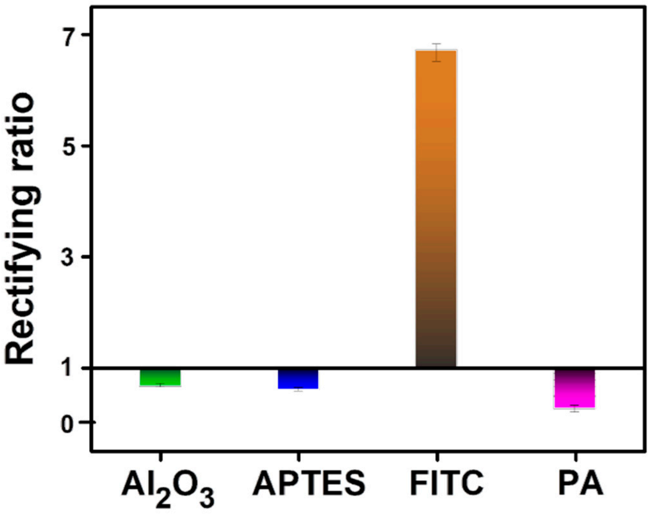

Figure S3. Ionic current rectifying ratio characteristics of the bare Al_2O_3, APTES, FITC, and PA nanochannels, representing the change in polarity and quantity of surface charges. (The rectifying ratio is a proportion of the absolute values of the ionic currents recorded at -2 V against the same absolute values of +2 V).

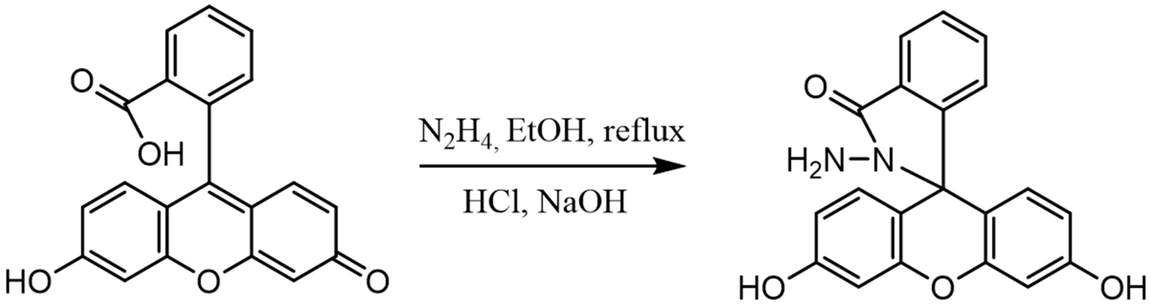

Figure S4. The synthesis of spiro form fluorescein hydrazide by the reaction of fluorescein with hydrazine hydrate.

Add 1.0 g (3.1 mmol) of fluorescein as well as 30 mL of absolute ethanol into a 50 mL flask and stir to obtain a yellow-red solution. At room temperature, slowly drop 1.55 g (31 mmol) of hydrazine hydrate into the system. Heating in a bath, reflux overnight at 85℃ to get a clarified yellow solution, on the same time of adopting thin layer chromatography (TLC) to monitor the reaction process. Cool and evaporate the solvent of the reaction solution in vacuo to give an orange-yellow porous primary product. Then, dis- solve the residue with 1 mol/L HCl aqueous solution upon stirring continuously to obtain a yellow turbid liquid. Slowly drop a slight excess of 1 mol/L NaOH aqueous solution into the above solution until the pH value of the system reaches 8-9, during which vast amounts of precipitate are formed. Finally, after adding distilled water and stirring for several hours, filter the precipitate and wash it with deionized water three times at room temperature. Dry in a vacuum oven for 24 h to obtain a grey or yellow crystalline powder. Yield: 80.6%.

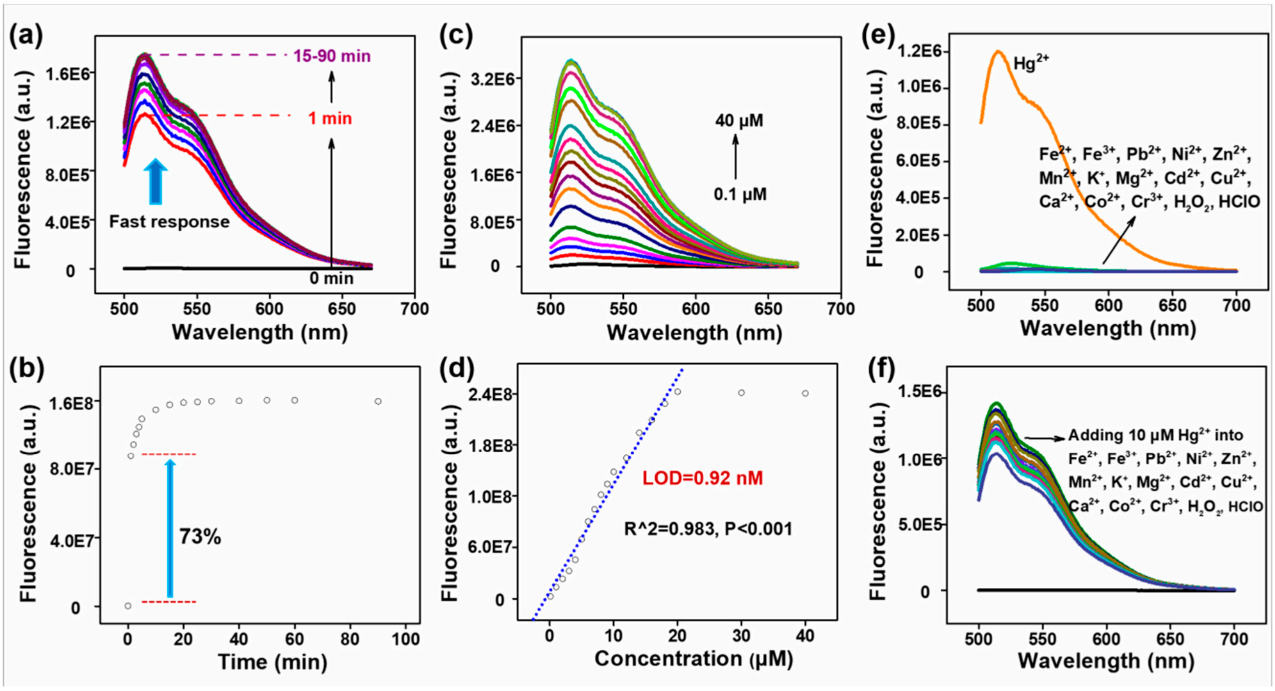

Figure S5. Liquid phase fluorescence detection. Fluorescence emission of fluorescein-N_2H_4 probe (10 μM) in ethanol solution (a and b) with the incremental response time in the presence of 1 equiv. Hg^{2+}, (c and d) with the incremental Hg^{2+} content (0-40 μM), (e) with 1 equiv. different ana- lytes, and (f) with 1 equiv. Hg^{2+} in the presence of 1 equiv. other different analytes. Excitation wavelength is 488 nm. The incubation time is 15 minutes.

The chemodosimeter predominantly highlights a quick response towards Hg^{2+} and the response already reached 73% (Figure S5a,b) within one minute. Then the fluorescence intensity of the probe solution is slightly increased until the response of Hg^{2+} is saturated at 15 minutes. More importantly, during the incubation time of 15-90 minutes, almost no fluorescence changes were observed. This trait indicated that the probe has good stability. Therefore, in this experiment, the incubation time of Hg^{2+} was set to 15 minutes for adequate reaction, if no specific instructions were given. It exhibits a sensi- tive "turn-on" fluorescent response toward Hg^{2+} with a detection limit of 0.92 nM (Fig- ure S5c,d). Furthermore, the probe demonstrates high specificity on Hg^{2+} and strong immunity from other metal ions interferences (Figure S5e,f). Only the presence of Hg^{2+} can cause the fluorescence intensity of the probe to increase greatly. In short, the spiro probe exhibited a good reaction rate, sensitivity, and selectivity.

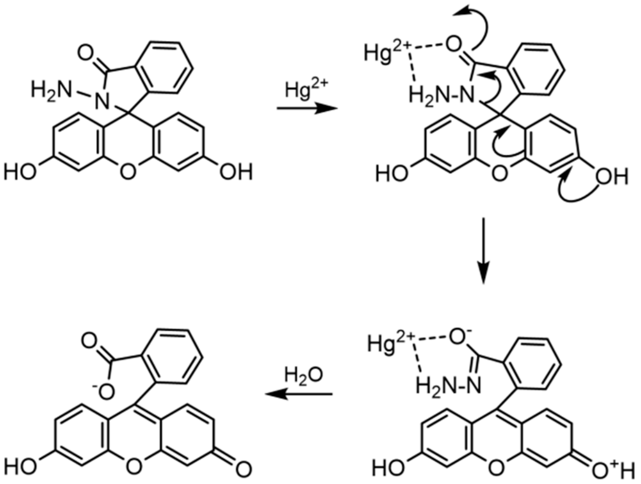

Figure S6. Schematic representation of possible mechanistic pathway of the catalytic reaction of Hg^{2+} on the non-fluorescence fluorescein-N_2H_4 probe to form highly-fluorescence fluorescein.

The free fluorescein-N_2H_4 probe is spirocyclic and nonfluorescent in the absence of Hg^{2+}. Upon the catalytic activity by Hg^{2+}, it coordinated with O and N atoms of hydrazone group simultaneously to form a metal-ligand complex intermediate that could rapidly induce reductive hydrolytic cleavage, ultimately leading to the formation of fluorescein that was open-ring and thus showed strong green fluorescence. Therefore, the presence of Hg^{2+} in the probe matrix could catalyze this hydrolysis procedure to achieve a transformation of probe conformation from fluorescein spriolactam to fluorescein.

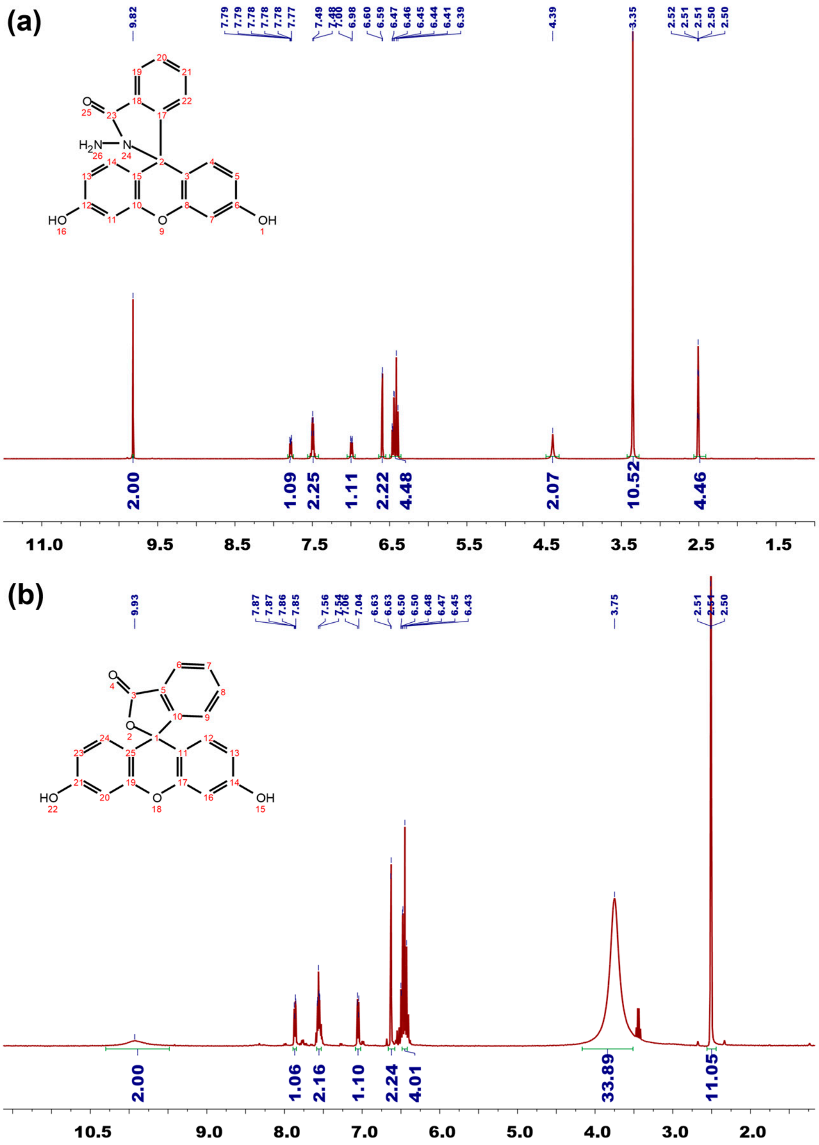

Figure S7. The ^{1}HNMR spectrum of the fluorescein-N_2H_4 probe in the (a) absence and (b) presence of Hg^{2+}. The action of mercury causes the amide bond cleavage and thus the N_2H_4 of the probe to be removed, restoring to fluorescein.

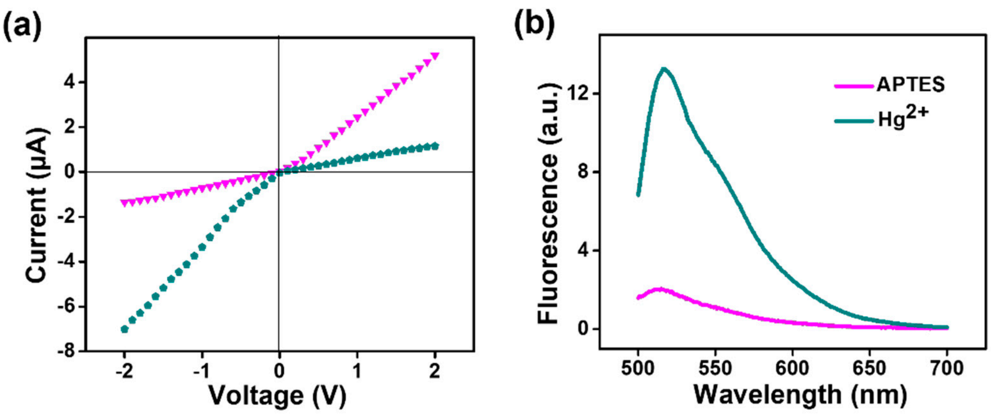

Figure S8. (a) Current and (b) fluorescence response to Hg^{2+} of PA nanochannels after being kept out of the light for months, suggesting that the functionalized PA nanosensor is stable.

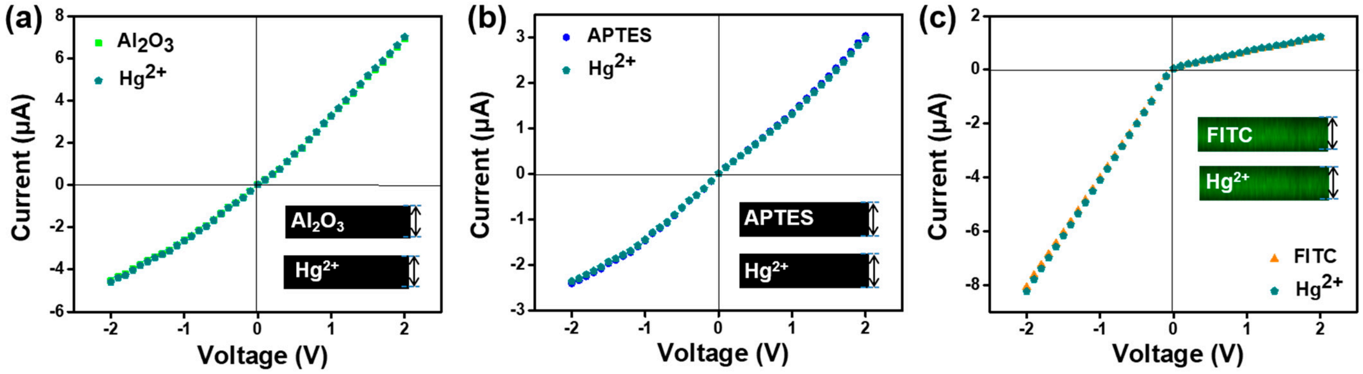

Figure S9. *I-V* properties of (a) Al_2O_3, (b) APTES, and (c) FITC nanochannels before and after Hg^{2+} stimulus, respectively, in each of which no significant current alterations were observed. The in- sets in Figure S9a-c are corresponding to several confocal imaging and the thickness of the nanochannels membrane was about 25 μm. The combined results of current and fluorescence demonstrated the effectiveness of the PA channel, instead of Al_2O_3, APTES, and FITC nanochan- nels, response to Hg^{2+}.

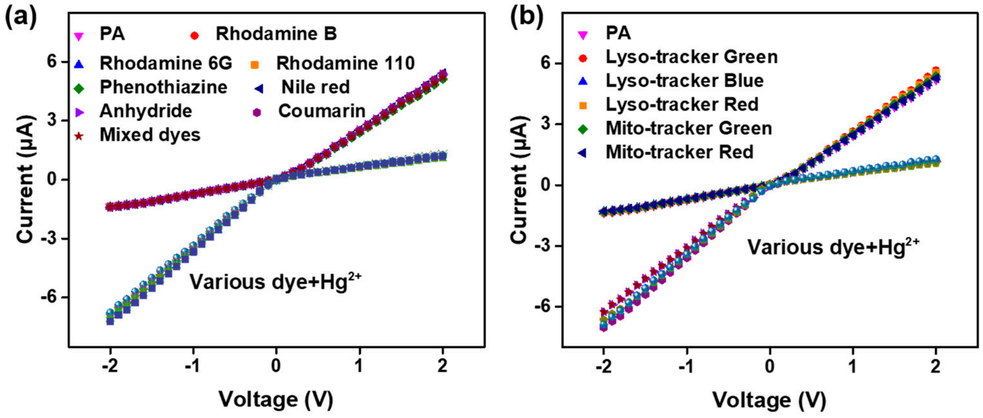

Figure S10. *I-V* curve response performance of PA nanochannels for indicating Hg^{2+} in a complex matrix containing diversified (a) chromophore dyes or (b) cell tracking dyes. The results showed the detection system has high selectivity and anti-interference capability.

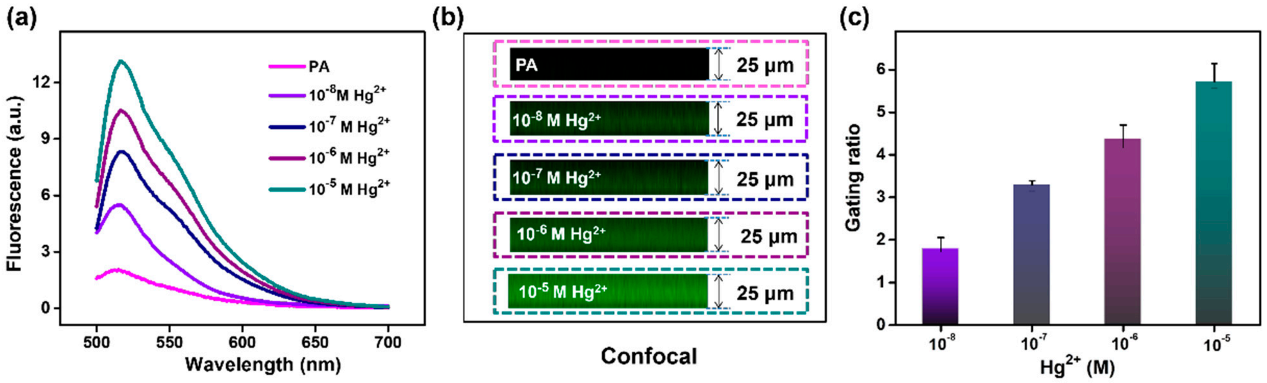

Figure S11. (a) Fluorescence spectra, (b) confocal imaging, and (c) corresponding fluorescence gating ratios of PA nanochannel system obtained in the presence of different concentrations of Hg^{2+} from 10^{-8} to 10^{-5} M.

The N_2H_4-modified nanochannels exhibited both OFF-state current at -2 V voltage and fluorescence due to the closing of the spiroring. But the addition of Hg^{2+} can effectively trigger the opening of the spiroring, resulting in the recovery of FITC channels and exposure of -COOH on the pore surface. In this case, the current- and fluorescence-gating are simultaneously switched to ON-state. As shown in Figure S11, increasing the concentration of Hg^{2+} from 10^{-8} to 10^{-5} M, both ionic current at -2 V and fluorescence intensity (Figure 7b and Figure S11a) are gradually raised, suggesting both current gating and fluorescence gating (Figure S11c) translating from OFF state to ON state and the successful creation of smart nanochannel sensor with build-in current and fluorescence dual gating.

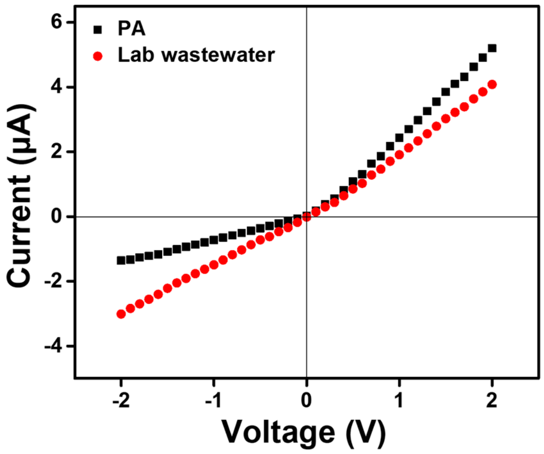

Figure S12. *I-V* results of PA nanochannels for determination of Hg^{2+} in lab wastewater samples.
